# Trehalose protects against oxidative stress by regulating the Keap1–Nrf2 and autophagy pathways

**DOI:** 10.1016/j.redox.2017.09.007

**Published:** 2017-09-20

**Authors:** Yuhei Mizunoe, Masaki Kobayashi, Yuka Sudo, Shukoh Watanabe, Hiromine Yasukawa, Daiki Natori, Ayana Hoshino, Arisa Negishi, Naoyuki Okita, Masaaki Komatsu, Yoshikazu Higami

**Affiliations:** a Laboratory of Molecular Pathology and Metabolic Disease, Faculty of Pharmaceutical Sciences, Tokyo University of Science, 2641 Noda, Chiba 278-8510, Japan; b Translational Research Center, Research Institute of Science and Technology, Tokyo University of Science, Noda 278-8510, Japan; c Department of Internal Medicine Research, Sasaki Institute, Sasaki Foundation, Tokyo 101-0062, Japan; d Department of Biochemistry, Niigata University Graduate School of Medical and Dental Sciences, Niigata 951-8510, Japan

**Keywords:** ATG, autophagy-related gene, DMEM, Dulbecco's Modified Eagle Medium, GFP, green fluorescent protein, HFD, high fat diet, Ho-1, heme oxygenase-1, Keap1, Kelch-like ECH-associated protein 1, LC3, microtubule-associated protein 1 light chain 3 (MAP1LC3), MCP1, monocyte chemoattractant protein-1, MEF, mouse embryonic fibroblast, mTOR, mechanistic target of rapamycin, Nqo1, nicotinamide adenine dinucleotide phosphate quinone dehydrogenase 1, NAFLD, non-alcoholic fatty liver disease, NASH, non-alcoholic steatohepatitis, Nrf2, nuclear factor (erythroid-derived 2)-like 2, RFP, red fluorescent protein, ROS, reactive oxygen species, RT-PCR, reverse transcription polymerase chain reaction, shRNA, small hairpin RNA, Trehalose, Oxidative stress, p62, Autophagy, Keap1–Nrf2 system, Antioxidant

## Abstract

Dysfunction of autophagy, which regulates cellular homeostasis by degrading organelles and proteins, is associated with pathogenesis of various diseases such as cancer, neurodegeneration and metabolic disease. Trehalose, a naturally occurring nontoxic disaccharide found in plants, insects, microorganisms and invertebrates, but not in mammals, was reported to function as a mechanistic target of the rapamycin (mTOR)-independent inducer of autophagy. In addition, trehalose functions as an antioxidant though its underlying molecular mechanisms remain unclear. In this study, we showed that trehalose not only promoted autophagy, but also increased p62 protein expression, in an autophagy-independent manner. In addition, trehalose increased nuclear translocation of nuclear factor (erythroid-derived 2)-like 2 (Nrf2) in a p62-dependent manner and enhance expression of its downstream antioxidant factors, heme oxygenase-1 (*Ho-1*) and nicotinamide adenine dinucleotide phosphate quinone dehydrogenase 1 (*Nqo1*). Moreover, treatment with trehalose significantly reduced amount of reactive oxygen species. Collectively, these results suggested that trehalose can function as a novel activator of the p62–Keap1/Nrf2 pathway, in addition to inducing autophagy. Therefore, trehalose may be useful to treat many chronic diseases involving oxidative stress and dysfunction of autophagy.

## Highlights

• A novel cellular protective mechanism of trehalose is identified.
• Trehalose activates autophagy.
• Trehalose increases p62 expression in an autophagy-independent manner.
• Trehalose-associated p62 upregulation activates the Keap1–Nrf2 pathway.
• Trehalose lowers oxidative stress via Nrf2-regulated antioxidant gene expression.

## 1 Introduction

Autophagy, a major proteolytic system for delivering cytoplasmic constituents to the lysosome for degradation by hydrolytic enzymes, plays an important role in cellular energy mobilization and homeostasis by clearing damaged organelles, aggregated proteins and pathogens [1]. This pathway is stimulated by diverse stresses, such as nutrient or energy depletion, oxidative stress, hypoxia, mitochondrial damage or pathogen infection [2]. Recent studies showed that dysfunction of autophagy contributes to pathology of various diseases including cancer, diabetes and neurodegenerative disorders [3]. Therefore, activators of autophagy might be effective therapeutics [4], [5]. Two proteins, LC3 and p62, have been widely used as markers for autophagic activity. Upon induction of autophagy, a cytosolic form of LC3 (LC3-I) is conjugated to phosphatidylethanolamine (known as LC3-II) localized on autophagosome membranes. Thus, LC3-II serves as a marker for autophagosome formation [6]. The p62 protein was identified as both a selective autophagy substrate and a cargo receptor for autophagic degradation of ubiquitinated targets [7]. As p62 protein accumulation was observed in parallel with suppression of autophagy, p62 is also commonly used as a marker of autophagic degradation [7], [8]. Furthermore, increased p62 regulates the stress-responsive transcription factor nuclear factor (erythroid-derived 2)-like 2 (Nrf2) [9], a key regulator of antioxidant and detoxification responses. Nrf2 binds to antioxidant responsive elements to induce expression of target genes, such as heme oxygenase-1 (*Ho-1*) and nicotinamide adenine dinucleotide phosphate quinone dehydrogenase 1 (*Nqo1*). Under physiological conditions, interaction with the E3 ubiquitin ligase Kelch-like ECH-associated protein 1 (Keap1) restricts localization of Nrf2 to the cytoplasm, where it is constantly degraded by the ubiquitin proteasome system. Under stress conditions, Keap1 gets oxidized with reactive oxygen species (ROS), then allows Nrf2 to translocate into nucleus [10], [11]. In addition to this classical pathway, p62 also enhances Nrf2 transcriptional activity through competitive interactions with Keap1 [9], [12].

Trehalose, a non-reducing disaccharide composed of two D-glucose units linked α-1,1, is present in many organisms, including bacteria, fungi, plants and invertebrates [13]. Trehalose is an inducer of the mechanistic target of rapamycin (mTOR)-independent autophagy [14], [15]. Indeed, several studies reported that trehalose excluded abnormally aggregated proteins by promoting autophagy [14], [15]. Additionally, recent studies showed that trehalose prevented obesity in mice [16], [17]. In these reports, administration of trehalose to obese mice mitigated insulin resistance by suppressing adipocyte hypertrophy and reducing insulin secretion. Moreover, trehalose treatment downregulated expression of monocyte chemoattractant protein-1 (MCP1) mRNA and upregulated serum adiponectin levels. However, mechanisms underlying the activities of trehalose are incompletely understood.

In this study, we investigated the pharmacological actions of trehalose by focusing on autophagy pathways and found that trehalose treatment promoted both p62 accumulation and autophagy. Finally, we showed that trehalose enhanced expression of antioxidant genes regulated by nuclear translocation of Nrf2, in a p62-dependent manner, proposing a novel mechanism of cellular protection against oxidative stress.

## 2 Material and methods

### 2.1 Cell lines and reagents

Mouse hepatoma Hepa1-6 cells, as well as wildtype autophagy-related protein 5 (*Atg5*^+/+^) and *Atg5*-knockout (*Atg5*^–/–^) mouse embryonic fibroblasts (MEFs), were purchased from RIKEN Bioresource Center (Ibaraki, Japan). Wildtype (*p62*^+/+^) and *p62*-knockout (*p62*^-/-^) MEFs were kindly provided by Dr. Toru Yanagisawa (University of Tsukuba, Ibaraki, Japan).

Trehalose dehydrate (204-18451), d(-)-sorbitol (194-03752), sucrose (193-00025), cycloheximide (CHX, 037-20991), chloroquine (CQ, 036-17972) and N-acetylcysteine (NAC, 015-05132) were from Wako (Tokyo, Japan). 1,1′-Dimethyl-4,4′-bipyridinium dichloride hydrate (paraquat, 36541) was from Sigma-Aldrich (St. Louis, MO, USA). D(+)-maltose (000-47122), isomaltose (I0231) and neotrehalose (α, β-trehalose, ON12751) were from Kishida Chemical (Osaka, Japan), Tokyo Kasei Kogyo (Tokyo, Japan), and Carbosynth (Compton, UK), respectively. Trehalose and other saccharides were dissolved in culture medium and filtered. CHX was dissolved in dimethylsulfoxide and then diluted into an aqueous solution containing less than 0.1% dimethylsulfoxide. Other reagents were dissolved in phosphate buffered saline (PBS).

### 2.2 Generation of Atg5-knockdown cells

Hepa1-6 cell lines expressing *Atg5* shRNA (shAtg5) and control luciferase shRNA (shLuc) were established using a NEPA21 electroporation system (Nepa Gene, Chiba, Japan) with pulse voltage = 150 V, pulse interval = 5 ms, pulse width = 50 ms and pulse number = 2.

Oligonucleotides for shAtg5 and shLuc control were chemically synthesized (Operon Biotechnology, Tokyo, Japan) as follows: shAtg5, 5′-GATAGCTTTCTTTATATTGGCTTCAAGAGAGCTAATATGAAGAAAGTTATCTTTTT-3′ and 5′- GCAAAAAGATAACTTTCTTCATATTAGCTCTCTTGAAGCCAATATAAAGAAAGCTATC-3′; and shLuc, 5′-GTACTGAGCCTGTTTGTGGAATTCAAGAGA TTTCACAAACGGGCTTAGTACTTTTT-3′ and 5′-CGAAAAAGTACTAAGCCCGTTTGTGAAATCTCTTGAATTCCACAAACAGGCTCAGTAC-3′. Annealed oligos were directly ligated into a Pme1- and BstB1-digested pMXs-neo-mU6 shRNA expression vector [18], [19]. After electroporation, cells were incubated in medium containing 1250 μg/mL G418 (Cayman Chemical, Ann Arbor, MI, USA) for 12 d of selection.

### 2.3 Establishment of Hepa1-6 cell lines overexpressing GFP-LC3 or mRFP-GFP-LC3

Plasmids encoding green fluorescent protein (GFP) fused to LC3 (GFP-LC3), or GFP and red fluorescent protein (RFP) tandemly tagged LC3 (mRFP-GFP-LC3), were obtained from Addgene (#21073 and #21074, respectively). To generate pMXs-AMNN-Puro-EGFP-LC3 and pMXs-AMNN-Puro-mRFP-GFP-LC3 plasmids, GFP-LC3 and mRFP-GFP-LC3 were each digested with *Nhe*I and *Eco*RI, and then subcloned into the same sites of the pIRES-neo3 vector. Subcloned vectors were then digested with *Eco*RV and *Not*I and cloned into the pMXs-AMNN-Puro vector at *Nru*I and *Not*I sites.

Hepa1-6 cell lines overexpressing GFP-LC3 or mRFP-GFP-LC3 were generated using retroviral infection as previously reported [19], [20]. Briefly, vectors were transfected into Plat-E cells with FuGENE^®^6 (Promega, Madison, WI, USA) according to the manufacturer's protocol. Virus-containing culture supernatants were collected 2 d after transfection and filtered through 0.22-μm filters (Millipore, Billerica, MA, USA). To obtain Hepa1-6 cell lines overexpressing GFP-LC3 or mRFP-GFP-LC3, Hepa1-6 cells were incubated with virus-containing medium for 2 d and then selected with 4 μg/mL puromycin for 5 d.

### 2.4 Establishment of MEFs overexpressing wildtype or mutant p62

Retroviral expression vectors for wildtype (WT) and mutant (S351E) *p62* were transfected into *p62*^-/-^ MEFs lacking endogenous p62. MEF overexpressing cell lines were generated using retroviral infection, as previously reported [20], [21] and described for Hepa1-6 cells. The promoter driving expression of p62 in this system is the retroviral 5'LTR promoter.

### 2.5 Cell culture and treatment

All cells were cultured in Dulbecco's Modified Eagle's Medium (DMEM) with high glucose, 10% fetal bovine serum (Sigma) and 1% penicillin/streptomycin (Sigma). Stable Atg5-knockdown or control cell lines (shAtg5 or shLuc) were incubated with medium containing 250 μg/mL G418. Other transfected cell lines were incubated with medium containing 0.8 mg/mL puromycin. All cultures were maintained in a 37 °C incubator with 95% air, 5% CO_2_.

Cells were treated for various times with different concentrations of trehalose or other sugars, harvested and analyzed by western blotting and real-time RT-PCR.

To analyze autophagic flux, Hepa1-6 cells were treated with trehalose in the absence or presence of 10 µM CQ. Additionally, to examine p62 degradation, Hepa1-6 shLuc/shAtg5 cells were pretreated with trehalose or CQ for 24 h and then medium was changed to medium containing 10 µM CHX for 0–240 min.

### 2.6 Western blotting and antibodies

Protein extraction and western blotting were performed as previously described [26]. Primary antibodies were as follows: LC3 (PM036, MBL, Nagoya, Japan), p62 (PM045, MBL), p-p62 (S351) (PM074, MBL), Nrf2 (H-300, sc-13032, Santa Cruz Biotechnology, Inc., CA, USA), β-actin (A1978, Sigma), Lamin B1 (PM064, MBL), α-tubulin (T6199, Calbiochem, Darmstadt, Germany). Secondary antibodies included horseradish peroxidase-conjugated F(ab′)_2_ fragments of goat anti-mouse IgG or anti-rabbit IgG (Jackson Immunoresearch, West Grove, PA, USA). Antibody-bound proteins were visualized with an LAS3000 Image Analyzer (Fujifilm, Tokyo, Japan) and data were analyzed using Multigauge software (Fujifilm).

### 2.7 Confocal imaging

Hepa1-6 cell lines overexpressing mRFP-GFP-LC3 were grown on a cover slide for 48 h and treated with PBS (control), 50 mM trehalose or 10 μM CQ for 24 h. These cells were fixed with 4% paraformaldehyde for 15 min and images were acquired on a FV1000 confocal microscope (Olympus, Tokyo, Japan) using a 63x objective.

Hepa1-6 cell lines overexpressing GFP-LC3 were grown on a cover slide for 48 h and treated with PBS (control), 50 mM trehalose or 10 μM CQ for 24 h. After staining with 100 nM Lysotracker Red DND-99 (Invitrogen, Carlsbad, CA, USA) for 15 min, the cells were fixed with 4% paraformaldehyde for 15 min. Images were acquired on a FV1000 confocal microscope (Olympus, Tokyo, Japan) using a 63x objective.

### 2.8 Isolation of nuclear and cytoplasmic fractions

Scraped and washed cell pellets were suspended in 300 µL buffer A (20 mM HEPES, pH 7.9, 3 mM MgCl_2_, 20 mM KCl, 0.68 M sucrose, 20% glycerol and 1% Triton X-100). After 10 min incubation on ice, cells were disrupted by pipetting and the mixture was centrifuged at 1300×*g* for 5 min. The supernatant fraction was then centrifuged at 1300×*g* for 10 min to remove any remaining nuclear debris. The supernatant from this step was used as the cytoplasmic protein extract, while the precipitate was resuspended in 800 µL buffer A and centrifuged at 1300×*g* for 4 min. The suspension was discarded and the precipitate was resuspended in 800 µL buffer A and centrifuged again at 1300×*g* for 4 min. The precipitate was obtained as the nuclear protein extract. Cytoplasmic, nuclear and total protein extracts were prepared using lysis buffer (50 mM Tris–HCl pH 6.8, 2% SDS and 5% glycerol).

### 2.9 RNA purification and RT-PCR

Total RNA was extracted from cells using a ReliaPrep RNA Miniprep System (Promega) according to the manufacturer's protocol. Purified RNA was subjected to reverse transcription with PrimeScript Reverse Transcriptase (Takara Bio, Otsu, Japan) and random hexamers (Takara). Quantitative real-time RT-PCR was performed using a CFX Connect™ RT-PCR System (Bio-Rad, Hercules, CA, USA) with SYBR® Premix ExTaq™II (Takara, RR820B), as previously described [20], [21]. Transcripts of *p62*, *Ho-1*, *Nqo1* and ribosomal protein S18 (*Rps18*) were amplified and *Rps18* was used for normalization. Primer sequences are shown in Table 1.

**Table 1:** Primer sequence.

*Ho-1*	Forward	5'-GAACTTTCAGAAGGGTCAGGTG-3'
	Reverse	5'-AGGGAAGTAGAGTGGGGCATAG-3'
	
*Nqo-1*	Forward	5'-CGAATCTGACCTCTATGCTATGAAC-3'
	Reverse	5'-GAACTGAAATATCACCAGGTCTGC-3'
	
*p62*	Forward	5'-TGGTGGGAACTCGCTATAAGTG-3'
	Reverse	5'-CCAAAGTGTCCATGTTTCAGC-3'
	
*MnSOD*	Forward	5'-CCCAAAGGAGAGTTGCTGGAG-3'
	Reverse	5'-CGACCTTGCTCCTTATTGAAGC-3'
	
*Gpx4*	Forward	5'-TCTGTGTAAATGGGGACGATG-3'
	Reverse	5'-AGGGGCACACACTTGTAGGG-3'
	
*Rps18*	Forward	5'-TGCGAGTACTCAACACCAACAT-3'
	Reverse	5'-CTTTCCTCAACACCACATGAGC-3'

### 2.10 ROS assay

Cellular ROS levels were measured using the cell permeable probe 5-(and-6)-chloromethyl-2',7'-dichlorodihydrofluorescein diacetate (CM-H_2_DCFDA, Thermo Fisher, Waltham, MA, USA). Cells were loaded with 10 μM H_2_DCFDA in DMEM (phenol red-free) for 1 h. After washing cells twice with DMEM, fluorescence was measured with an Envision 2104 Multilabel reader (Perkin Elmer, Waltham, MA, USA).

### 2.11 Statistical analysis

Statistical analysis was performed using the Tukey–Kramer test or Student's *t*-test (for comparison of two means). Data are presented as means ± standard deviation (S.D.). A *p*-value of less than 0.05 was considered significant.

## 3 Results

### 3.1 Trehalose increased p62 expression by an autophagy-independent mechanism

**Fig. 1:**
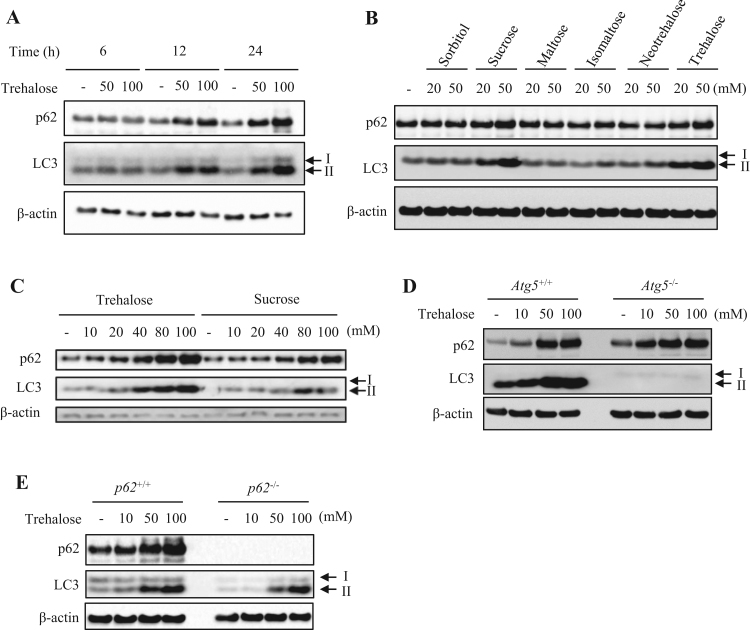
Trehalose affects autophagy marker protein expression. (A) Hepa1-6 cells were treated with the indicated concentrations of trehalose for 6, 12 or 24 h. (B) Hepa1-6 cells were treated with 20 or 50 mM of various saccharides for 24 h. (C) Hepa1-6 cells were treated with the indicated concentrations of trehalose or sucrose for 24 h. (D) *Atg5*^+/+^ or *Atg5*^-/-^ mouse embryonic fibroblasts (MEFs) were treated with the indicated concentrations of trehalose for 24 h. (E) *p62*^+/+^ or *p62*^-/-^ MEFs were treated with the indicated concentrations of trehalose for 24 h. β-Actin was used as a loading control. Data are representative of two independent experiments.

To analyze the effect of trehalose on autophagy in Hepa1-6 cells, we investigated expression of LC3-I, LC3-II and p62 proteins by western blotting. Treatment with trehalose increased both LC3-II and p62 levels in concentration- and time-dependent manners (Fig. 1A). In contrast, these levels were not affected by treatment with sugar alcohol (sorbitol) or other disaccharides formed from two units of glucose (maltose, isomaltose and neotrehalose; Fig. 1B). When sucrose (composed of glucose and fructose) was applied, both LC3-II and p62 levels were increased, but the changes were not as robust as those induced by trehalose (Fig. 1C and Supplementary Fig. 1). We next assessed whether increased p62 expression was attributable to suppression of autophagic degradation, using MEFs derived from wildtype (*Atg5*^+/+^) and *Atg5*-knockout (*Atg5*^-/-^) mice. Treatment with trehalose increased both LC3-II and p62 levels in wildtype MEFs and Hepa1-6 cells (Fig. 1C and D). Similarly, trehalose upregulated p62 expression in autophagy-deficient *Atg5*^-/-^ MEFs (Fig. 1D) and LC3-II expression in *p62*^-/-^ MEFs (Fig. 1E).

### 3.2 Trehalose degraded p62 protein by an autophagic process

**Fig. 2:**
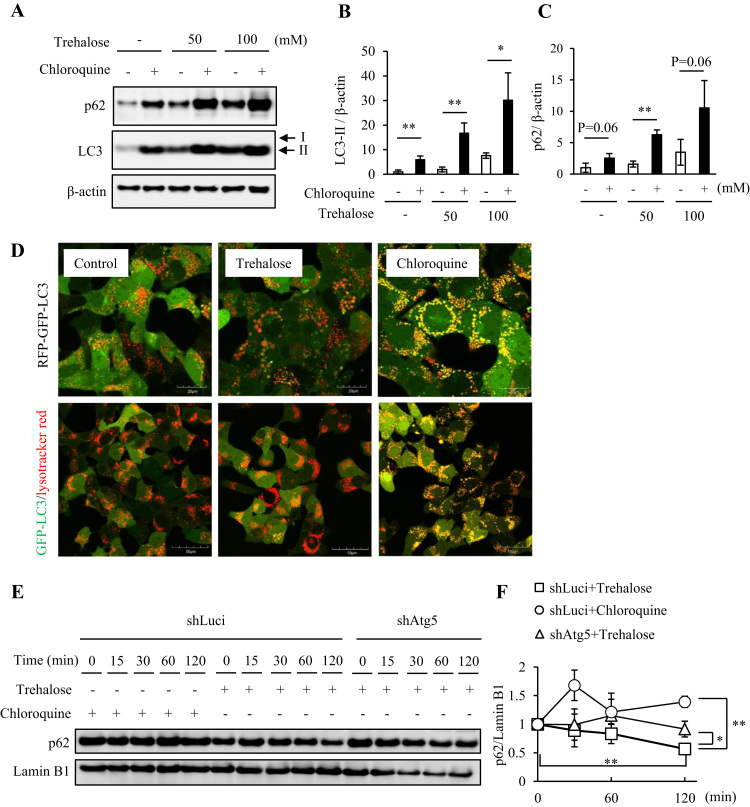
Trehalose did not inhibit autophagic flux but accelerated p62 turnover. (A–C) Hepa1-6 cells were treated for 24 h with 50 or 100 mM trehalose, with or without chloroquine (CQ), while untreated cells were used as controls. Total cell lysates were analyzed by western blotting using anti-p62, LC3 and β-actin antibodies (A) and bands were quantified (B, C). β-Actin was used as a loading control. Representative images and quantitative data (n = 4) are shown. Values are means ± SD. Differences between values were analyzed by Student's *t*-test. Statistical significance shown as *p < 0.05, **p < 0.01. (D) Hepa1-6/RFP-GFP-LC3 cells were treated with 50 mM trehalose or 10 μM CQ for 8 h (upper panel). Hepa1-6 cells were treated with 50 mM trehalose or 10 μM CQ for 8 h and stained with LysoTracker Red for 30 min (lower panels). Images were acquired by confocal fluorescence laser microscopy. Scale bars are 20 µm and 50 µm, respectively. (E, F) Hepa1-6 sh*Luc* (control)/sh*Atg5* cells were pretreated with 50 mM trehalose or 10 μM CQ for 24 h and then treated with 200 μM cycloheximide (CHX) for the indicated times. Total cell lysates were analyzed by western blotting using anti-p62 and LaminB1 antibodies and quantified. LaminB1 was used as a loading control. Representative images are shown (E). The quantitative data (n = 3) is shown as relative to the values at time 0 min for each cell type and under each experimental condition (F). Differences between values were analyzed by Student's *t*-test. Statistical significance shown as *p < 0.05, **p < 0.01.

To further examine the effects of trehalose on autophagic p62 degradation, we applied an LC3-II turnover assay that has recently been widely used to analyze autophagic flux [22]. In this assay, application of CQ, an inhibitor of lysosomal acidification and autophagic clearance, increased LC3-II and p62 accumulation in trehalose-treated cells, compared with in untreated cells (Fig. 2A–C). To investigate autophagic flux in more detail, we introduced mRFP-GFP-LC3, which enabled discrimination between early autophagosomes exhibiting dual red and green fluorescence and autolysosomes exhibiting only red fluorescence [23]. While CQ significantly induced yellow puncta, trehalose treatment yielded red puncta similar to those in PBS treated cells, indicating that trehalose did not suppress autophagy (Fig. 2D). Moreover, to evaluate lysosomal clearance in detail, Hepa1-6 cells stably transfected with GFP-LC3 were stained with LysoTracker Red (LTR). CQ markedly induced co-localization of GFP-LC3 puncta with LTR-labeled lysosomes. In contrast, trehalose treated cells exhibited many LTR-positive lysosomes and fewer GFP-LC3 puncta, compared with PBS treated control cells (Fig. 2D), suggesting that autophagic clearance was not attenuated in trehalose treated cells.

We next examined the time-dependence of p62 degradation after treatment with CHX, an inhibitor of protein biosynthesis. In control (shLuc) Hepa1-6 cells, trehalose treatment continuously decreased p62 protein levels, while CQ treatment did not. However, trehalose treatment significantly lowered p62 protein levels, compared with CQ treatment for 2 h. Moreover, the trehalose induced decrease in p62 protein levels was significantly suppressed in shAtg5-treated Hepa1-6 cells (genetic inhibition of autophagy processes), compared with in controls (Fig. 2E and F). This suggested that trehalose treatment did not impair autophagic machinery, including lysosomal clearance systems.

### 3.3 Trehalose increased p62 protein expression and promoted Nrf2 nuclear translocation

**Fig. 3:**
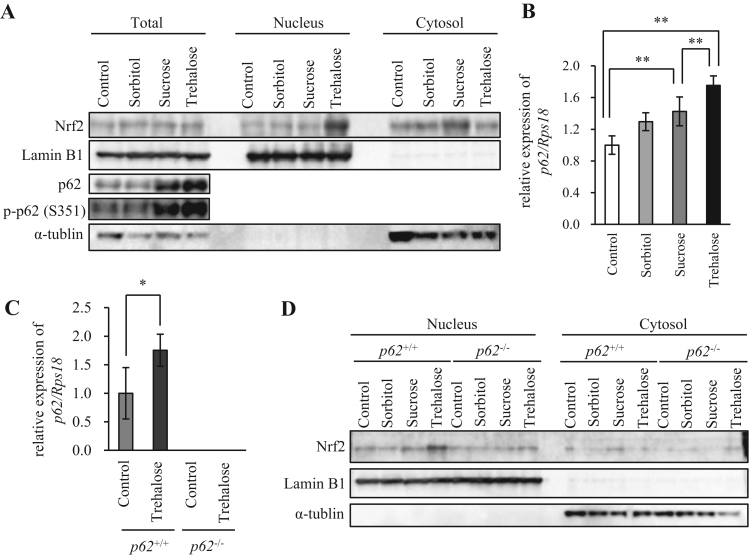
Trehalose enhanced the effects of p62 expression and promoted Nrf2 nuclear translocation. (A) Hepa1-6 cells were treated with 50 mM sorbitol, sucrose or trehalose for 24 h, while untreated cells were used as a control. Total, nuclear and cytoplasmic protein extracts were prepared and analyzed by western blotting using anti-Nrf2, p62, p-p62 (S351), LaminB1 and α-tubulin antibodies. LaminB1 and α-tubulin were used as the loading controls for nuclear and cytoplasmic protein extracts, respectively. (B) Hepa1-6 cells were treated with 50 mM sorbitol, sucrose, or trehalose for 24 h and harvested, while untreated cells were used as a control. *p62* mRNA expression was analyzed by real-time RT-PCR (n = 4). Data were normalized against *Rps18* expression (n = 4). Values are means ± SD. Differences among values were analyzed by the Tukey-Kramer method with *p < 0.05, **p < 0.01. (C) *p62*^+/+^ or *p62*^-/-^ mouse embryonic fibroblasts (MEFs) were treated with 50 mM trehalose for 24 h and harvested. Expression of *p62* mRNA was analyzed by real-time RT-PCR (n = 4). Data were normalized against *Rps18* (n = 4). Values are means ± SD. Differences between values were analyzed by Student's *t*-test. Statistical significance shown as *p < 0.05, **p < 0.01. (D) *p62*^+/+^ or *p62*^-/-^ MEFs were treated with 50 mM sorbitol, sucrose or trehalose for 24 h. Untreated cells were used as a control. Nuclear and cytoplasmic protein extracts were prepared and analyzed by western blotting using anti-Nrf2, LaminB1 and α-tubulin antibodies. LaminB1 and α-tubulin were used as the loading controls for nuclear and cytoplasmic protein extracts, respectively. Data are representative of two independent experiments.

Trehalose treatment did not attenuate autophagic flux, but it greatly increased p62 protein levels. Hence, we investigated whether trehalose induced increases in p62 protein affected the Keap1–Nrf2 pathway in Hepa1-6 cells. The p62 protein, when phosphorylated at serine residue 351 (S351), showed a higher affinity for Keap1 and promoted Nrf2 nuclear translocation [9], [24]. Both sucrose and trehalose increased total p62 protein and the S351-phosphorylated form of p62 (p-p62) protein, and the effect of trehalose was more significant than that of sucrose in Hepa1-6 cells. Trehalose treatment increased nuclear Nrf2 markedly, compared with sorbitol or sucrose (Fig. 3A). In agreement with this result, *p62* mRNA expression was more highly induced by trehalose than by sorbitol or sucrose (Fig. 3B). To confirm the p62-dependence of trehalose induced Nrf2 nuclear translocation, we analyzed the effects of trehalose and compared them with those of sorbitol and sucrose in *p62*^+/+^ and *p62*^-/-^ MEFs. Trehalose also increased *p62* mRNA expression in *p62*^+/+^ MEFs (Fig. 3C). In *p62*^+/+^ MEFs, trehalose markedly increased Nrf2 expression within the nuclear protein extract, whereas the other sugars did not (Fig. 3D). Moreover, trehalose-induced increases in nuclear Nrf2 protein were not observed in *p62*^-/-^ MEFs (Fig. 3D). These results indicated that Nrf2 nuclear translocation induced by trehalose treatment was dependent on p62 protein expression.

### 3.4 Trehalose induced Nrf2 nuclear translocation predominantly via upregulated p62 expression

**Fig. 4:**
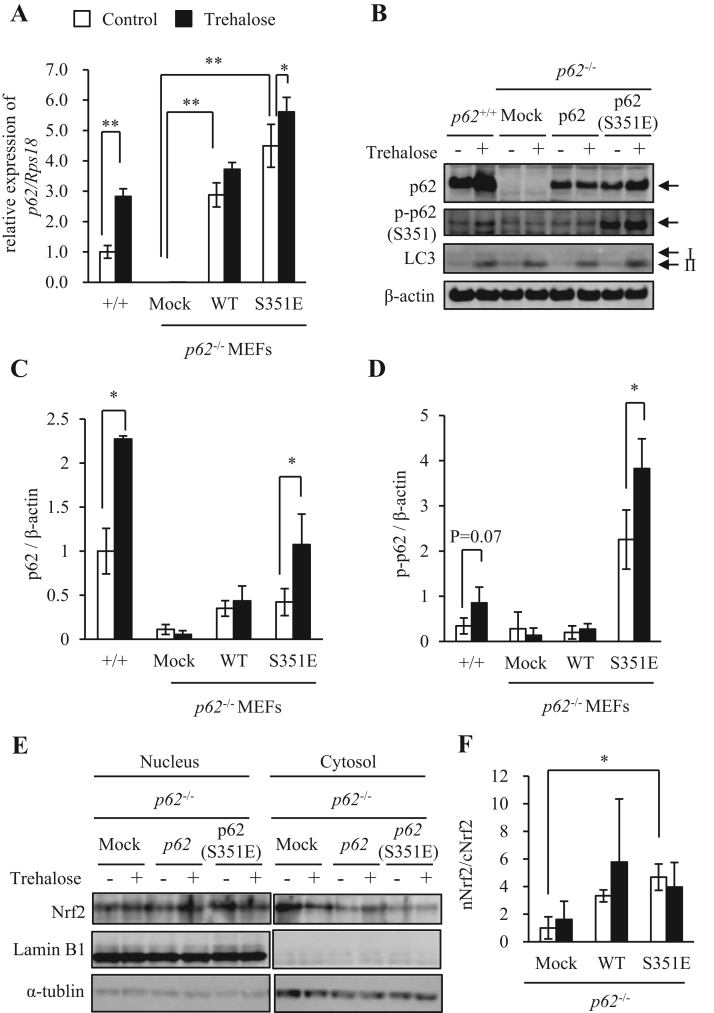
Trehalose induced Nrf2 nuclear translocation, predominantly through upregulation of *p62* mRNA and protein expression. (A) *p62*^+/+^ mouse embryonic fibroblasts (MEFs) and *p62*^*-*/-^ MEF stable cell lines were treated with 50 mM trehalose for 24 h and harvested. mRNA expression of *p62* was analyzed by real-time RT-PCR (n = 4). Data were normalized against *Rps18* (n = 4). Values are means ± SD. Differences among values were analyzed by the Tukey-Kramer method with *p < 0.05, **p < 0.01. (B–D) *p62*^*-*/-^ MEFs were stably transfected with mock, wildtype p62 or p62 S351E mutant. The *p62*^+/+^ MEFs and *p62*^-/-^ MEF stable cell lines were treated with 50 mM trehalose for 24 h and analyzed by western blotting using p62, p-p62 (S351E), LC3 and β-actin antibodies. Quantitative data for p62 are shown in (C). Quantitative data for p-p62 are shown as values excluding the background signals observed in mock *p62*^*-*/-^ MEFs (D). β-Actin was used as a loading control. Representative images and quantitative data (n = 4) are shown. Values are means ± SD. Differences between values were analyzed by Student's *t*-test. Statistical significance shown as *p < 0.05, **p < 0.01. (E-F) *p62*^*-*/-^ MEF stable cell lines were treated with 50 mM trehalose for 24 h. Nuclear and cytoplasmic protein extracts were prepared and analyzed by western blotting using anti-Nrf2, LaminB1 and α-tubulin antibodies. LaminB1 and α-tubulin were used as the loading controls for nuclear and cytoplasmic protein extracts, respectively. Representative images (E) and quantitative date (F) in Ratios between nuclear Nrf2 (nNrf2) and cytoplasmic Nrf2 (cNrf2) (n=3) are shown. Values are means ± SD. Differences between values were analyzed by Student's *t*-test. Statistical significance shown as *p < 0.05, **p < 0.01.

It was reported that oxidative stress promoted nuclear translocation of Nrf2 protein by enhancing p62 phosphorylation in an mTOR-dependent manner [9], [24]. As shown in Fig. 3A, trehalose treatment, compared with sucrose treatment, increased p-p62 protein. This finding suggested two interpretations: (1) trehalose may directly activate certain kinases that phosphorylate p62 protein, and/or (2) trehalose may upregulate expression of either *p62* mRNA or total p62 protein. To distinguish between these possibilities, we generated *p62*^-/-^ MEFs in which wildtype (WT) or S351E mutant (mimic of constitutive p62 phosphorylation) p62 was stably expressed. Notably, as *p62* mRNA expression levels were not physiologically regulated in these p62 stable cell lines, we could evaluate only effects of trehalose that were independent of transcriptional regulation. In *p62*^-/-^ MEFs transfected with either WT p62 or S351E mutant, trehalose induced upregulation of *p62* mRNA expression was not as great as in *p62*^+/+^ MEFs (Fig. 4A). In addition, trehalose did not exhibit much effect on total and p-p62 protein levels in these p62 revertants (Fig. 4B, C and D). However, p-p62 bands were also observed in mock *p62*^-/-^ MEFs in this analysis (Fig. 4B). Thus, we regarded these bands as nonspecific signals because total p62 protein was not present in mock *p62*^-/-^ MEFs. Henceforth, we calculated the quantity of p-p62, based on the band intensity, and subtracted the values determined in mock *p62*^-/-^ MEFs as background signals (Fig. 4D). Trehalose-induced nuclear translocation of Nrf2, represented by an Nrf2 nuclear/cytoplasm ratio (nNrf2/cNrf2), was not increased in *p62*^-/-^ MEFs transfected with either WT p62 or S351E mutant (Fig. 4E and F). These results supported the hypothesis that trehalose, unlike oxidative stress, did not significantly contribute to phosphorylation of p62 protein. Collectively, these findings indicated that trehalose could induce nuclear translocation of Nrf2 via upregulated expression of p62 mRNA and protein.

### 3.5 Trehalose increased antioxidant gene expression and reduced paraquat-induced ROS

**Fig. 5:**
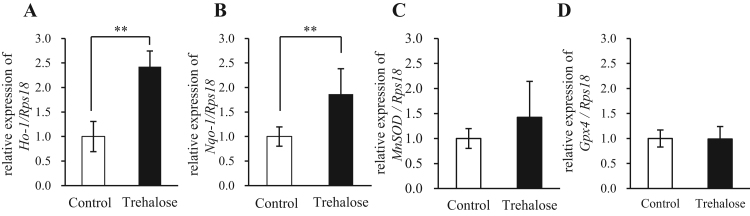
Trehalose exerted effects on the Keap1–Nrf2 pathway by altering p62 protein expression. (A-D) Hepa1-6 cells were treated with 50 mM trehalose for 24 h and harvested, while untreated cells served as a control. Expression of *Ho-1* (A), *Nqo1* (B), *MnSOD* (C) and *Gpx4* (D) mRNA analyzed by real-time RT-PCR (n = 4). Data were normalized to *Rps18* expression (n = 4). Values are means ± SD. Differences between values were analyzed by Student's *t*-test. Statistical significance shown as *p < 0.05, **p < 0.01.

**Fig. 6:**
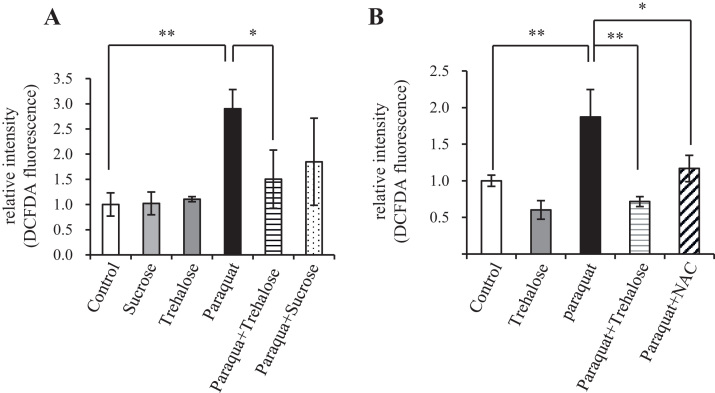
Trehalose suppressed oxidative stress. (A, B) Hepa1-6 cells were pretreated with 50 mM trehalose or sucrose, or 10 mM N-acetylcysteine (NAC) for 24 h before incubation with 2 mM paraquat for 15 h. Intracellular ROS levels were analyzed with CM-H_2_DCFDA (n = 5). Values indicate means ± SD. Differences among values were analyzed by the Tukey-Kramer method with *p < 0.05, **p < 0.01.

We examined the ability of trehalose to upregulate expression of downstream gene targets of Nrf2, upon its increased nuclear translocation. Indeed, trehalose significantly increased mRNA expression levels of the Nrf2 target genes *Ho-1* and *Nqo1* (Fig. 5A and B). In contrast, the mRNA levels of *MnSOD* and *Gpx4* were not changed by trehalose treatment (Fig. 5C and D). Paraquat is commonly used as an experimental ROS generator because it modulates mitochondrial function and increases mitochondrial oxidative damage [25], [26]. Therefore, we analyzed effects of trehalose on paraquat-induced ROS, using 2′,7′-dichlorodihydrofluorescein diacetate (H_2_DCFDA) fluorescence as an indicator of ROS. Pretreatment with trehalose, but not sucrose, suppressed paraquat-induced H_2_DCFDA fluorescence (Fig. 6A). Moreover, the antioxidant activity of trehalose was comparable to that of NAC, which is commonly used as an antioxidant (Fig. 6B).

## 4 Discussion

Trehalose was previously reported to activate mTOR-independent autophagy in COS-7 cells, PC12 cells and MEFs [4], [5]. We showed here that trehalose also activated autophagy flux in Hepa1-6 cells and MEFs. Recently, however, certain reports suggested that trehalose suppressed autophagy flux in H4 cells, HeLa cells and MEFs [27], [28]. While we do not know the exact reasons for these discrepancies, they may have resulted from differences in experimental conditions. Moreover, while it is well known that trehalose functions as an antioxidant [29], its targets have yet to be determined. Regardless, suppression of autophagy and exposure to various stresses, including hydrogen peroxide and proteasome inhibitors, increased p62 protein expression [30], [31]. Increased expression of phosphorylated p62 forms inhibited Keap1–Nrf2 interactions, thereby activating nuclear translocation and transcriptional activity of Nrf2 to promote transcription of Nrf2 target genes, such as detoxification enzymes and antioxidant molecules [9], [24]. In our study, we showed that trehalose protected against oxidative stress by regulating the Keap1–Nrf2 pathway in an autophagy-independent manner. As trehalose upregulated expression of p62 transcripts in Hepa1-6 cells, differentiated 3T3-L1 adipocytes (data not shown) and even autophagy-deficient *Atg5*^-/-^ MEFs, we considered that trehalose may have not only activated autophagy, but also induced transcription of Nrf2 target genes by upregulating p62 and, subsequently, inhibiting Keap1-Nrf2 interactions, leading to protection against oxidative stress. Indeed, Giorgetti et al. [32] recently reported that trehalose increased p62 mRNA and protein levels in mouse motor neuron-like cells. Such findings also supported our data.

A recent report demonstrated that sucrose also activated autophagy in MEFs [33]. Our findings suggested that trehalose and sucrose equally activated autophagy. However, trehalose promoted Nrf2 nuclear translocation by upregulating total p62 and p-p62 protein, leading to transcriptional activation of Nrf2 target genes, while sucrose did not. Our study showed that pretreatment with trehalose or NAC strongly reduced paraquat-induced ROS (Fig. 6B). Sucrose pretreatment also slightly decreased paraquat-induced ROS without affecting levels of Nrf2 nuclear translocation and antioxidant gene expression. Collectively, these data suggested that the antioxidant effects of sucrose might be caused in part by autophagy, as autophagy acts to regulate intracellular ROS levels by clearing damaged mitochondria [34]. Notably, as sucrose, maltose, isomaltose, neotrehalose and trehalose are similar disaccharides formed by the linkage of two glucose molecules, we did not consider the observed effects of trehalose on autophagy and Nrf2 nuclear translocation to involve changes in osmotic pressure.

The phosphorylated form of p62, which is more prone to interact with Keap1 than the unphosphorylated form, induced Nrf2 nuclear translocation more effectively [24]. Therefore, we hypothesized that trehalose promoted Nrf2 nuclear translocation through enhanced p62 phosphorylation. However, in *p62*^-/-^ MEFs transfected with wildtype *p62*, trehalose failed to increase p62 phosphorylation and Nrf2 nuclear translocation, suggesting that trehalose may have promoted *p62* transcription by certain activating transcriptional factors (Fig. 3, Fig. 4). Transcription factor EB (TFEB) is known to upregulate expression of lysosome- and autophagy-related genes, including p62, to activate the autophagy-lysosome pathway [35], [36]. Indeed, trehalose activated TFEB transcriptional activity [37]. Nrf2 also activated p62 gene transcription, indicating that transcriptional regulation of p62 expression can be enhanced by a positive feedback loop [38]. In addition, trehalose might upregulate p62 protein levels through other p62 phosphorylation-dependent mechanisms because trehalose induced p62 protein expression in *p62*^-/-^ MEFs transfected with S351E. Generally, it is known that trehalose works as a universal stabilizer of protein conformation and function [39]. Moreover, it has been also reported that trehalose treatment stabilizes the mRNA by acting on the enzymes [40]. Considering these reports, there is a possibility that trehalose may function as an mRNA stabilizer, possibly resulting in an increase of p62 mRNA. In our study, we unexpectedly observed that p-p62 levels in *p62*^-/-^ MEFs rescued with WT *p62* were not elevated compared with in mock transfected MEFs. This may have resulted from the low kinase activity required to phosphorylate p62 protein in MEFs. Therefore, further experiments are required to clarify the mechanisms underlying upregulation of p62 by trehalose.

Generally, trehalose is hydrolyzed into two molecules of glucose by trehalase. Expression of trehalase is not ubiquitous but, instead, is restricted to the kidney brush border membrane and the intestinal villi membranes in mammals [41]. Hence, it is unlikely that enzymatic hydrolysis of trehalose into glucose occurs in the other cells/tissues. Moreover, it was reported that trehalose ingestion did not rapidly increase blood glucose levels in healthy individuals. It was shown that orally administered trehalose rapidly accumulated in peripheral circulation in mice [42], [43], [44]. These reports suggested that trehalose is not substantially metabolized and acts directly on the peripheral tissues/cells in the form of a disaccharide. Interestingly, Mayer et al. demonstrated that trehalose was taken into the cell via SLC2A8 (GLUT8) protein, a homolog of the drosophila trehalose transporter-1 (Tret1), and induced autophagy in a GLUT8-AMPK-dependent manner [44]. It is unknown whether trehalose enhanced the antioxidant capacity via SLC2A8 protein, but it is important that this issue be investigated in the future.

Nrf2 activation may be involved in cytoprotection of normal cells, as well as in growth and survival of tumor cells [24], [45]. For example, several reports demonstrated the ability of naturally occurring Nrf2 activators, including curcumin, to protect against carcinogenesis [46], [47]. Curcumin promoted Nrf2 activity by disrupting the interaction between Keap1 and Nrf2 in renal epithelial cells [48] and improved glucose intolerance and insulin resistance in HFD-induced obese mice [49]. Oltipraz, a synthetic Nrf2 activator, improved both insulin resistance and inflammation induced by HFD [50]. Pharmacological Nrf2 activators only modestly increased Nrf2 activity and decreased intracellular ROS levels, with beneficial effects against obesity, compared with the effects of genetic modifications [51]. In contrast, "constitutive" Nrf2 activation and dysregulation of the Keap1–Nrf2 pathway [52], as well as accumulation of p-p62 and high Nrf2 expression [53], were observed in certain cancer cells. Moreover, constitutive activation of Nrf2 induced by Keap1 gene knockdown promoted high fat diet (HFD)-induced fatty liver and glucose intolerance in Leptin-null (Lep^o**b**/**ob**^) mice [54]. Although little is known about the anti-obesity mechanisms of trehalose [16], [17], [55], taken together, these results suggested that trehalose may suppress pathologies related to obesity by activating Nrf2 and autophagy. In fact, it was already reported that trehalose improved metabolic parameters [56] and prevented hepatic steatosis in obese *Atg7*^*+/−*^ mice, through attenuation of autophagy [42]. These previous findings, along with our study, provided evidence that trehalose may have valuable therapeutic potential for cancer prevention and anti-obesity.

## 5 Conclusions

We provided evidence that trehalose not only activated autophagy, but also upregulated p62 expression. Moreover, trehalose upregulated antioxidant gene expression via enhanced nuclear translocation of Nrf2, in a p62-dependent manner, leading to suppression of oxidative stress. Thus, our results proposed a novel antioxidant target of action for trehalose.

## Funding

This work was supported by Grants-in-Aid for Scientific Research (C) (No. 19590396) and Challenging Exploratory Research (No. 26670193) from the Japan Society for the Promotion of Science and the MEXT-Supported Program for the Strategic Research Foundation at Private Universities, 2014–2018.

## Appendix A Supplementary material

Supplementary material: Fig. 1. (A–C) Hepa1-6 cells were treated with 100 mM sorbitol, sucrose, or trehalose for 24 h and harvested, while untreated cells were used as a control. Total cell lysates were analyzed by western blotting using anti-p62, LC3 and β-actin antibodies (A) and bands were quantified (B, C). β-Actin was used as a loading control. Representative images and quantitative data (n = 3) are shown. Values are means ± SD. Differences between values were analyzed by Tukey-Kramer method with **p* < 0.05, ***p* < 0.01.
