# Supplementary figures and images for: Trehalose protects against oxidative stress by regulating the Keap1–Nrf2 and autophagy pathways
Source: Redox Biol. 2017 Sep 20;15:115–24. doi: 10.1016/j.redox.2017.09.007 (PMC5730428; doi:10.1016/j.redox.2017.09.007)

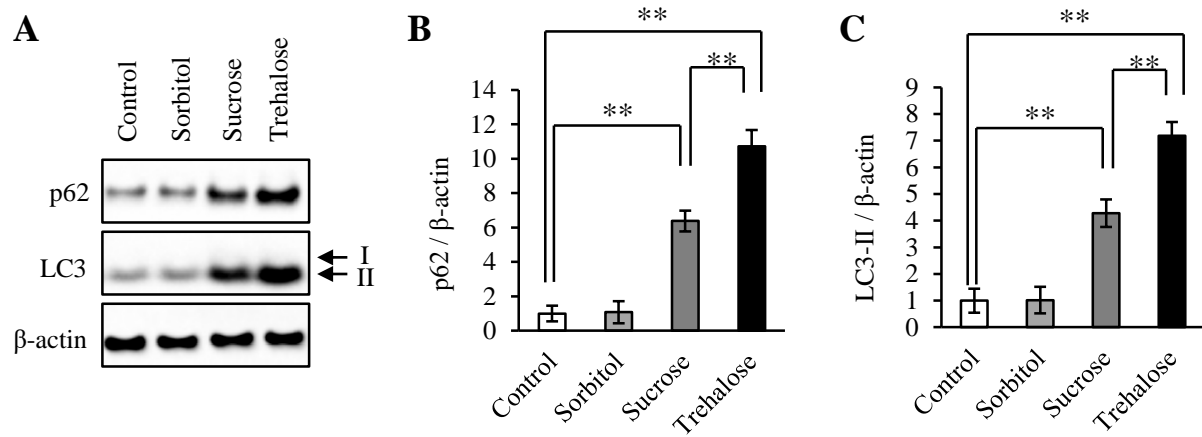

**Supplementary Figure 1**

Supplement: Supplementary file 1 — Supplementary material: Fig. 1. (A–C) Hepa1-6 cells were treated with 100 mM sorbitol, sucrose, or trehalose for 24 h and harvested, while untreated cells were used as a control. Total cell lysates were analyzed by western blotting using anti-p62, LC3 and β-actin antibodies (A) and bands were quantified (B, C). β-Actin was used as a loading control. Representative images and quantitative data (n = 3) are shown. Values are means ± SD. Differences between values were analyzed by Tukey-Kramer method with *p < 0.05, **p < 0.01. [file mmc1.pdf]
